# Supplementary figures and images for: Leveraging dynamic stability to infer regulation in protein-protein interaction networks: A study of infectious vulnerability in COPD
Source: PLoS One. 2025 Sep 5;20(9):e0326062. doi: 10.1371/journal.pone.0326062 (PMC12412946; doi:10.1371/journal.pone.0326062)

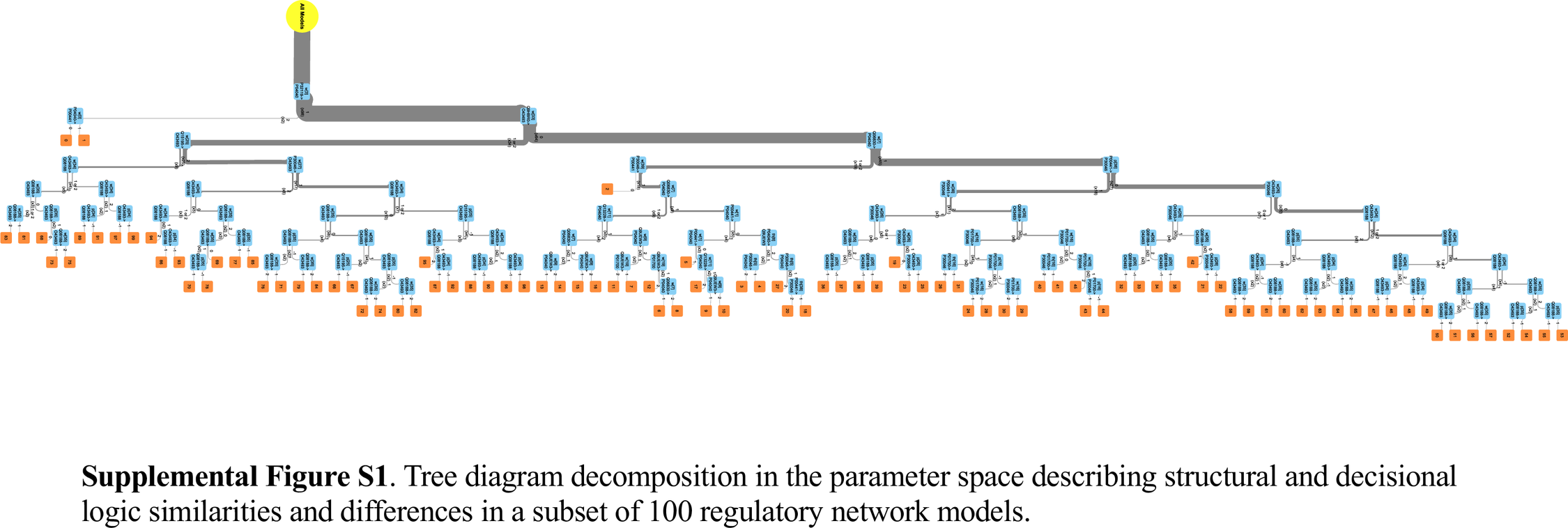

Supplement: S1 Fig — (TIF) [file pone.0326062.s001.tif]
